# Bis{benzyl 2-[4-(4-meth­oxy­phen­yl)butan-2-yl­idene]hydrazinecarbodithio­ato-κ^2^ *N* ^2^,*S*}nickel(II)

**DOI:** 10.1107/S1600536812019289

**Published:** 2012-05-05

**Authors:** Ming-Yueh Tan, Thahira Begum S. A. Ravoof, Mohamed Ibrahim Mohamed Tahir, Karen A. Crouse, Edward R. T. Tiekink

**Affiliations:** a Department of Chemistry, Universiti Putra Malaysia, 43400 Serdang, Malaysia; b Department of Chemistry, University of Malaya, 50603 Kuala Lumpur, Malaysia

## Abstract

The complete mol­ecule of the title complex, [Ni(C_19_H_21_N_2_OS_2_)_2_], is generated by the application of twofold symmetry. The Ni^II^ atom is *N*,*S*-chelated by two hydrazinecarbodithio­ate ligands, which provide an N_2_S_2_ donor set that defines a distorted square-planar geometry, the S atoms being approximately *cis*. The conformation of the chelate ring is an envelope with the Ni^II^ atom being the flap atom. The dihedral angle between the least-squares planes through the chelate rings = 30.10 (6)°. Supra­molecular chains propagated by glide symmetry along the *c* axis and mediated by C—H⋯N contacts feature in the crystal packing.

## Related literature

For background to the coordination chemistry of hydrazine carbodithio­ates, see: Khoo *et al.* (2005 ▶); Chan *et al.* (2008 ▶); Manan *et al.* (2012 ▶). For related syntheses, see Hossain *et al.* (1996 ▶).
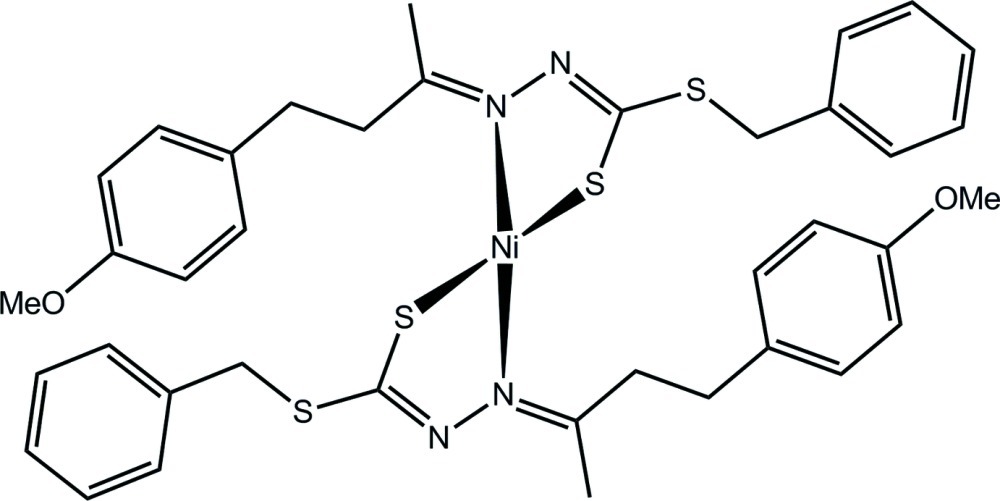


## Experimental

### 

#### Crystal data

[Ni(C_19_H_21_N_2_OS_2_)_2_]
*M* *_r_* = 773.71
Monoclinic, 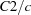

*a* = 24.0691 (6) Å
*b* = 12.5847 (2) Å
*c* = 12.4179 (3) Å
β = 101.857 (2)°
*V* = 3681.16 (14) Å^3^
*Z* = 4
Mo *K*α radiation
μ = 0.79 mm^−1^
*T* = 150 K
0.23 × 0.14 × 0.08 mm

#### Data collection

Agilent Xcalibur Eos Gemini diffractometer
Absorption correction: multi-scan (*CrysAlis PRO*; Agilent, 2011 ▶) *T*_min_ = 0.89, *T*_max_ = 0.94
46243 measured reflections
4223 independent reflections
3750 reflections with *I* > 2σ(*I*)
*R*_int_ = 0.041

#### Refinement

*R*[*F* ^2^ > 2σ(*F* ^2^)] = 0.030
*wR*(*F* ^2^) = 0.079
*S* = 1.01
4223 reflections
224 parameters
H-atom parameters constrained
Δρ_max_ = 0.80 e Å^−3^
Δρ_min_ = −0.45 e Å^−3^

### 

Data collection: *CrysAlis PRO* (Agilent, 2011 ▶); cell refinement: *CrysAlis PRO*; data reduction: *CrysAlis PRO*; program(s) used to solve structure: *SHELXS97* (Sheldrick, 2008 ▶); program(s) used to refine structure: *SHELXL97* (Sheldrick, 2008 ▶); molecular graphics: *ORTEP-3* (Farrugia, 1997 ▶) and *DIAMOND* (Brandenburg, 2006 ▶); software used to prepare material for publication: *publCIF* (Westrip, 2010 ▶).

## Supplementary Material

Crystal structure: contains datablock(s) global, I. DOI: 10.1107/S1600536812019289/hg5219sup1.cif

Structure factors: contains datablock(s) I. DOI: 10.1107/S1600536812019289/hg5219Isup2.hkl

Additional supplementary materials:  crystallographic information; 3D view; checkCIF report

## Figures and Tables

**Table:** 

Ni—N2	1.9220 (13)
Ni—S1	2.1543 (4)

**Table:** 

N2—Ni—S1	85.92 (4)
N2^i^—Ni—S1	156.70 (4)
S1^i^—Ni—S1	93.66 (2)
N2—Ni—N2^i^	103.49 (8)

Symmetry code: (i) 
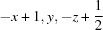
.

**Table 2:** Hydrogen-bond geometry (Å, °)

*D*—H⋯*A*	*D*—H	H⋯*A*	*D*⋯*A*	*D*—H⋯*A*
C10—H10*C*⋯N1^ii^	0.98	2.62	3.575 (2)	166

Symmetry code: (ii) 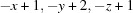.

**Fig. 1.**
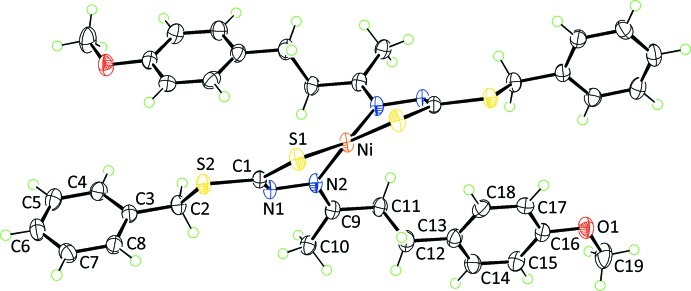
The molecular structure of (I) showing the atom-labelling scheme and displacement ellipsoids at the 50% probability level.

**Fig. 2.**
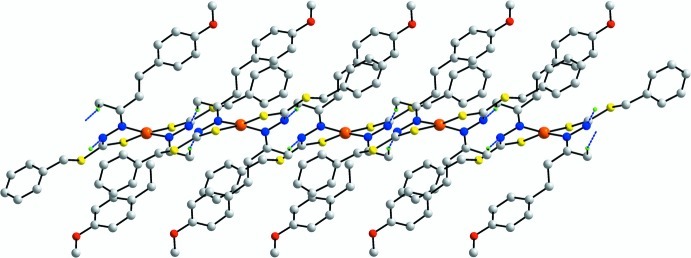
A view of the supramolecular chain in (I) mediated by C—H···N interactions, shown as blue dashed lines. Hydrogen atoms not involved in these interactions are omitted.

## supplementary crystallographic information

### Comment

In our on-going investigations to expand the scope of hydrazinecarbodithioate derivatives, their coordination chemistry and bio-activities (Khoo *et al.*, 2005; Chan *et al.*, 2008; Manan *et al.*, 2012), the title complex, (I), was synthesized and characterized crystallographically.

In (I), Fig. 1, the Ni^II^ atom exists within a distorted square planar *cis*-N_2_S_2_ donor set defined by two *N*,*S*-chelating hydrazinecarbodithioate ligands, Table 1. The five-membered chelate ring in non-planar (r.m.s. = 0.239 Å) but has an envelope conformation with the Ni atom being the flap atom. A measure of the distortion from the ideal square planar geometry is the dihedral angle of 30.10 (6)° formed between the least-squares planes through the chelate rings. The coordination geometry in (I) resembles that seen in a closely related analogue (Chan *et al.*, 2008).

The hydrazinecarbodithioate ligand is twisted about the N1—N2 bond with the C1—N1—N2—C9 torsion angle being 146.88 (14)°. The dihedral angle between the terminal benzene and phenyl rings is 89.37 (8)°, indicating an almost orthogonal relationship. The methoxy group is co-planar with the benzene ring to which it is connected as seen in the value of the C19—O1—C16—C15 torsion angle of -1.9 (3)°.

The most prominent feature of the crystal packing is the formation of supramolecular chains mediated by C—H···N contacts, Fig. 2 and Table 1. The chains are propagated by glide symmetry along the *c* axis.

### Experimental

4-(4-Methoxyphenyl)butan-2-one (1.70 g, 0.01 mol) in absolute ethanol (20 ml) was added to *S*-benzyldithiocarbazate (1.98 g, 0.01 mol, prepared as previously described by Hossain *et al.*, 1996) dissolved in hot absolute ethanol (20 ml). The mixture was heated (~340 K) while being stirred for half an hour and then cooled to room temperature. The Schiff base thus formed was filtered and dried *in vacuo* over anhydrous silica gel. A combination of hot absolute ethanol solutions of the Schiff base (0.36 g, 10 mmol, in 30 ml) and nickel(II) acetate tetrahydrate (0.12 g, 5 mmol, in 10 ml) was stirred at ~340 K for half an hour. The mixture was cooled to room temperature and a green precipitate formed. Dark-green crystals were obtained from its acetonitrile solution after one week. Yield 66%, *M*.pt: 417 K.

### Refinement

Carbon-bound H-atoms were placed in calculated positions (C—H = 0.95 to 0.99 Å) and were included in the refinement in the riding model approximation with *U*_iso_(H) set to 1.2 to 1.5*U*_equiv_(C).

### Crystal data

**Table:**

[Ni(C_19_H_21_N_2_OS_2_)_2_]	*F*(000) = 1624
*M**_r_* = 773.71	*D*_x_ = 1.396 Mg m^−^^3^
Monoclinic, *C*2/*c*	Mo *K*α radiation, λ = 0.71073 Å
Hall symbol: -C 2yc	Cell parameters from 17769 reflections
*a* = 24.0691 (6) Å	θ = 2.1–28.8°
*b* = 12.5847 (2) Å	µ = 0.79 mm^−^^1^
*c* = 12.4179 (3) Å	*T* = 150 K
β = 101.857 (2)°	Block, dark-green
*V* = 3681.16 (14) Å^3^	0.23 × 0.14 × 0.08 mm
*Z* = 4	

### Data collection

**Table:**

Agilent Xcalibur Eos Gemini diffractometer	4223 independent reflections
Radiation source: fine-focus sealed tube	3750 reflections with *I* > 2σ(*I*)
Graphite monochromator	*R*_int_ = 0.041
Detector resolution: 16.1952 pixels mm^-1^	θ_max_ = 27.5°, θ_min_ = 2.4°
ω scans	*h* = −31→31
Absorption correction: multi-scan (*CrysAlis PRO*; Agilent, 2011)	*k* = −16→16
*T*_min_ = 0.89, *T*_max_ = 0.94	*l* = −16→16
46243 measured reflections	

### Refinement

**Table:**

Refinement on *F*^2^	Primary atom site location: structure-invariant direct methods
Least-squares matrix: full	Secondary atom site location: difference Fourier map
*R*[*F*^2^ > 2σ(*F*^2^)] = 0.030	Hydrogen site location: inferred from neighbouring sites
*wR*(*F*^2^) = 0.079	H-atom parameters constrained
*S* = 1.01	*w* = 1/[σ^2^(*F*_o_^2^) + (0.0408*P*)^2^ + 4.0091*P*] where *P* = (*F*_o_^2^ + 2*F*_c_^2^)/3
4223 reflections	(Δ/σ)_max_ = 0.001
224 parameters	Δρ_max_ = 0.80 e Å^−^^3^
0 restraints	Δρ_min_ = −0.45 e Å^−^^3^

### Special details

**Table:**

Geometry. All s.u.'s (except the s.u. in the dihedral angle between two l.s. planes) are estimated using the full covariance matrix. The cell s.u.'s are taken into account individually in the estimation of s.u.'s in distances, angles and torsion angles; correlations between s.u.'s in cell parameters are only used when they are defined by crystal symmetry. An approximate (isotropic) treatment of cell s.u.'s is used for estimating s.u.'s involving l.s. planes.
Refinement. Refinement of *F*^2^ against ALL reflections. The weighted *R*-factor *wR* and goodness of fit *S* are based on *F*^2^, conventional *R*-factors *R* are based on *F*, with *F* set to zero for negative *F*^2^. The threshold expression of *F*^2^ > 2σ(*F*^2^) is used only for calculating *R*-factors(gt) *etc*. and is not relevant to the choice of reflections for refinement. *R*-factors based on *F*^2^ are statistically about twice as large as those based on *F*, and *R*- factors based on ALL data will be even larger.

### Fractional atomic coordinates and isotropic or equivalent isotropic displacement parameters (Å^2^)

**Table:**

	*x*	*y*	*z*	*U*_iso_*/*U*_eq_	
Ni	0.5000	0.72365 (2)	0.2500	0.02370 (9)	
S1	0.519791 (18)	0.60653 (3)	0.37871 (3)	0.02860 (10)	
S2	0.569791 (17)	0.65527 (3)	0.60936 (3)	0.02596 (10)	
O1	0.21872 (5)	0.90781 (10)	−0.20500 (10)	0.0370 (3)	
N1	0.51555 (6)	0.79888 (10)	0.47242 (10)	0.0241 (3)	
N2	0.48562 (6)	0.81821 (10)	0.36271 (10)	0.0241 (3)	
C1	0.53185 (6)	0.70112 (12)	0.48301 (12)	0.0230 (3)	
C2	0.57260 (7)	0.77439 (12)	0.69281 (13)	0.0270 (3)	
H2A	0.5936	0.8310	0.6626	0.032*	
H2B	0.5336	0.8002	0.6918	0.032*	
C3	0.60211 (7)	0.74882 (13)	0.80923 (12)	0.0253 (3)	
C4	0.65150 (7)	0.80300 (13)	0.85687 (14)	0.0295 (3)	
H4	0.6669	0.8548	0.8156	0.035*	
C5	0.67827 (8)	0.78137 (14)	0.96470 (15)	0.0346 (4)	
H5	0.7116	0.8194	0.9973	0.041*	
C6	0.65693 (8)	0.70515 (14)	1.02490 (14)	0.0340 (4)	
H6	0.6758	0.6900	1.0982	0.041*	
C7	0.60778 (8)	0.65067 (14)	0.97798 (14)	0.0322 (4)	
H7	0.5928	0.5985	1.0194	0.039*	
C8	0.58062 (7)	0.67255 (13)	0.87054 (13)	0.0289 (3)	
H8	0.5470	0.6350	0.8386	0.035*	
C9	0.44507 (7)	0.88749 (12)	0.35373 (13)	0.0252 (3)	
C10	0.42911 (7)	0.94234 (13)	0.44989 (14)	0.0301 (3)	
H10A	0.4409	0.8987	0.5160	0.045*	
H10B	0.3879	0.9527	0.4356	0.045*	
H10C	0.4481	1.0115	0.4610	0.045*	
C11	0.40945 (7)	0.91090 (13)	0.24206 (13)	0.0274 (3)	
H11A	0.4308	0.8915	0.1848	0.033*	
H11B	0.4012	0.9880	0.2357	0.033*	
C12	0.35317 (8)	0.84817 (16)	0.22310 (15)	0.0379 (4)	
H12A	0.3617	0.7713	0.2322	0.045*	
H12B	0.3316	0.8691	0.2795	0.045*	
C13	0.31683 (7)	0.86730 (14)	0.10992 (14)	0.0300 (3)	
C14	0.27371 (7)	0.94139 (14)	0.09299 (14)	0.0335 (4)	
H14	0.2669	0.9818	0.1536	0.040*	
C15	0.23988 (7)	0.95834 (13)	−0.01120 (14)	0.0324 (4)	
H15	0.2103	1.0097	−0.0214	0.039*	
C16	0.24986 (7)	0.89951 (13)	−0.09951 (13)	0.0283 (3)	
C17	0.29304 (7)	0.82389 (14)	−0.08368 (14)	0.0301 (3)	
H17	0.2998	0.7830	−0.1440	0.036*	
C18	0.32596 (7)	0.80872 (14)	0.01994 (14)	0.0321 (4)	
H18	0.3555	0.7573	0.0302	0.039*	
C19	0.17215 (9)	0.98027 (18)	−0.22191 (17)	0.0487 (5)	
H19A	0.1452	0.9589	−0.1766	0.073*	
H19B	0.1531	0.9796	−0.2997	0.073*	
H19C	0.1861	1.0520	−0.2011	0.073*	

### Atomic displacement parameters (Å^2^)

**Table:**

	*U*^11^	*U*^22^	*U*^33^	*U*^12^	*U*^13^	*U*^23^
Ni	0.03115 (16)	0.01813 (14)	0.01918 (14)	0.000	−0.00098 (11)	0.000
S1	0.0414 (2)	0.01877 (18)	0.02279 (19)	0.00211 (15)	−0.00002 (16)	0.00056 (14)
S2	0.0314 (2)	0.02222 (19)	0.02147 (19)	0.00228 (15)	−0.00117 (15)	0.00338 (14)
O1	0.0373 (7)	0.0417 (7)	0.0275 (6)	0.0092 (5)	−0.0035 (5)	0.0002 (5)
N1	0.0269 (7)	0.0232 (6)	0.0191 (6)	0.0003 (5)	−0.0025 (5)	0.0011 (5)
N2	0.0289 (7)	0.0199 (6)	0.0202 (6)	0.0010 (5)	−0.0028 (5)	0.0008 (5)
C1	0.0235 (7)	0.0234 (7)	0.0205 (7)	−0.0023 (6)	0.0005 (6)	0.0019 (6)
C2	0.0317 (8)	0.0234 (7)	0.0236 (8)	0.0004 (6)	0.0003 (6)	0.0011 (6)
C3	0.0270 (8)	0.0256 (7)	0.0221 (7)	0.0032 (6)	0.0022 (6)	−0.0010 (6)
C4	0.0298 (8)	0.0275 (8)	0.0301 (8)	−0.0014 (6)	0.0034 (7)	−0.0002 (6)
C5	0.0324 (9)	0.0339 (9)	0.0325 (9)	0.0014 (7)	−0.0047 (7)	−0.0055 (7)
C6	0.0400 (10)	0.0360 (9)	0.0223 (8)	0.0094 (7)	−0.0020 (7)	−0.0031 (7)
C7	0.0376 (9)	0.0338 (9)	0.0255 (8)	0.0042 (7)	0.0076 (7)	0.0049 (7)
C8	0.0268 (8)	0.0315 (8)	0.0267 (8)	−0.0002 (7)	0.0018 (6)	0.0019 (6)
C9	0.0265 (8)	0.0201 (7)	0.0266 (8)	−0.0025 (6)	−0.0004 (6)	0.0006 (6)
C10	0.0301 (8)	0.0261 (8)	0.0314 (8)	0.0035 (6)	0.0004 (7)	−0.0020 (6)
C11	0.0278 (8)	0.0246 (7)	0.0268 (8)	0.0021 (6)	−0.0011 (6)	0.0041 (6)
C12	0.0351 (9)	0.0445 (10)	0.0298 (9)	−0.0110 (8)	−0.0033 (7)	0.0076 (8)
C13	0.0274 (8)	0.0325 (8)	0.0278 (8)	−0.0073 (7)	0.0001 (6)	0.0031 (7)
C14	0.0359 (9)	0.0332 (9)	0.0300 (8)	−0.0042 (7)	0.0035 (7)	−0.0073 (7)
C15	0.0317 (9)	0.0278 (8)	0.0350 (9)	0.0036 (7)	0.0004 (7)	−0.0032 (7)
C16	0.0269 (8)	0.0293 (8)	0.0261 (8)	−0.0025 (6)	−0.0004 (6)	0.0004 (6)
C17	0.0269 (8)	0.0358 (9)	0.0280 (8)	0.0015 (7)	0.0071 (6)	−0.0021 (7)
C18	0.0231 (8)	0.0357 (9)	0.0363 (9)	0.0021 (7)	0.0033 (7)	0.0034 (7)
C19	0.0449 (11)	0.0524 (12)	0.0401 (11)	0.0189 (9)	−0.0116 (9)	−0.0004 (9)

### Geometric parameters (Å, º)

**Table:**

Ni—N2	1.9220 (13)	C8—H8	0.9500
Ni—N2^i^	1.9220 (13)	C9—C11	1.502 (2)
Ni—S1^i^	2.1543 (4)	C9—C10	1.496 (2)
Ni—S1	2.1543 (4)	C10—H10A	0.9800
S1—C1	1.7391 (15)	C10—H10B	0.9800
S2—C1	1.7435 (15)	C10—H10C	0.9800
S2—C2	1.8157 (16)	C11—C12	1.544 (2)
O1—C16	1.3731 (19)	C11—H11A	0.9900
O1—C19	1.427 (2)	C11—H11B	0.9900
N1—C1	1.290 (2)	C12—C13	1.514 (2)
N1—N2	1.4250 (17)	C12—H12A	0.9900
N2—C9	1.296 (2)	C12—H12B	0.9900
C2—C3	1.509 (2)	C13—C14	1.379 (2)
C2—H2A	0.9900	C13—C18	1.393 (2)
C2—H2B	0.9900	C14—C15	1.396 (2)
C3—C8	1.390 (2)	C14—H14	0.9500
C3—C4	1.392 (2)	C15—C16	1.384 (2)
C4—C5	1.388 (2)	C15—H15	0.9500
C4—H4	0.9500	C16—C17	1.393 (2)
C5—C6	1.379 (3)	C17—C18	1.379 (2)
C5—H5	0.9500	C17—H17	0.9500
C6—C7	1.388 (3)	C18—H18	0.9500
C6—H6	0.9500	C19—H19A	0.9800
C7—C8	1.387 (2)	C19—H19B	0.9800
C7—H7	0.9500	C19—H19C	0.9800
			
N2—Ni—S1^i^	156.70 (4)	C9—C10—H10A	109.5
N2^i^—Ni—S1^i^	85.92 (4)	C9—C10—H10B	109.5
N2—Ni—S1	85.92 (4)	H10A—C10—H10B	109.5
N2^i^—Ni—S1	156.70 (4)	C9—C10—H10C	109.5
S1^i^—Ni—S1	93.66 (2)	H10A—C10—H10C	109.5
N2—Ni—N2^i^	103.49 (8)	H10B—C10—H10C	109.5
C1—S1—Ni	93.55 (5)	C9—C11—C12	110.92 (13)
C1—S2—C2	101.05 (7)	C9—C11—H11A	109.5
C16—O1—C19	116.58 (14)	C12—C11—H11A	109.5
C1—N1—N2	110.11 (12)	C9—C11—H11B	109.5
C9—N2—N1	114.89 (13)	C12—C11—H11B	109.5
C9—N2—Ni	126.78 (11)	H11A—C11—H11B	108.0
N1—N2—Ni	117.34 (9)	C13—C12—C11	112.44 (14)
N1—C1—S1	125.18 (12)	C13—C12—H12A	109.1
N1—C1—S2	119.97 (12)	C11—C12—H12A	109.1
S1—C1—S2	114.84 (9)	C13—C12—H12B	109.1
C3—C2—S2	109.02 (11)	C11—C12—H12B	109.1
C3—C2—H2A	109.9	H12A—C12—H12B	107.8
S2—C2—H2A	109.9	C14—C13—C18	118.25 (15)
C3—C2—H2B	109.9	C14—C13—C12	121.48 (16)
S2—C2—H2B	109.9	C18—C13—C12	120.26 (16)
H2A—C2—H2B	108.3	C13—C14—C15	121.46 (16)
C8—C3—C4	119.14 (15)	C13—C14—H14	119.3
C8—C3—C2	121.16 (14)	C15—C14—H14	119.3
C4—C3—C2	119.70 (15)	C16—C15—C14	119.30 (16)
C5—C4—C3	120.01 (16)	C16—C15—H15	120.4
C5—C4—H4	120.0	C14—C15—H15	120.4
C3—C4—H4	120.0	O1—C16—C15	124.44 (15)
C6—C5—C4	120.57 (16)	O1—C16—C17	115.58 (14)
C6—C5—H5	119.7	C15—C16—C17	119.96 (15)
C4—C5—H5	119.7	C18—C17—C16	119.69 (16)
C5—C6—C7	119.78 (16)	C18—C17—H17	120.2
C5—C6—H6	120.1	C16—C17—H17	120.2
C7—C6—H6	120.1	C17—C18—C13	121.34 (16)
C8—C7—C6	119.88 (16)	C17—C18—H18	119.3
C8—C7—H7	120.1	C13—C18—H18	119.3
C6—C7—H7	120.1	O1—C19—H19A	109.5
C7—C8—C3	120.61 (16)	O1—C19—H19B	109.5
C7—C8—H8	119.7	H19A—C19—H19B	109.5
C3—C8—H8	119.7	O1—C19—H19C	109.5
N2—C9—C11	119.22 (14)	H19A—C19—H19C	109.5
N2—C9—C10	123.63 (14)	H19B—C19—H19C	109.5
C11—C9—C10	117.06 (14)		
			
N2—Ni—S1—C1	−20.20 (7)	C6—C7—C8—C3	−0.1 (3)
N2^i^—Ni—S1—C1	94.98 (11)	C4—C3—C8—C7	0.2 (2)
S1^i^—Ni—S1—C1	−176.85 (6)	C2—C3—C8—C7	−179.21 (15)
C1—N1—N2—C9	146.88 (14)	N1—N2—C9—C11	−177.81 (13)
C1—N1—N2—Ni	−22.50 (16)	Ni—N2—C9—C11	−9.6 (2)
N2^i^—Ni—N2—C9	61.33 (12)	N1—N2—C9—C10	−1.3 (2)
S1^i^—Ni—N2—C9	−50.50 (19)	Ni—N2—C9—C10	166.88 (12)
S1—Ni—N2—C9	−140.27 (13)	N2—C9—C11—C12	98.22 (18)
N2^i^—Ni—N2—N1	−130.72 (12)	C10—C9—C11—C12	−78.50 (18)
S1^i^—Ni—N2—N1	117.45 (11)	C9—C11—C12—C13	−178.33 (15)
S1—Ni—N2—N1	27.68 (10)	C11—C12—C13—C14	−96.4 (2)
N2—N1—C1—S1	0.54 (19)	C11—C12—C13—C18	84.4 (2)
N2—N1—C1—S2	179.85 (10)	C18—C13—C14—C15	−0.2 (3)
Ni—S1—C1—N1	16.98 (14)	C12—C13—C14—C15	−179.42 (16)
Ni—S1—C1—S2	−162.36 (8)	C13—C14—C15—C16	0.0 (3)
C2—S2—C1—N1	1.00 (15)	C19—O1—C16—C15	−1.9 (3)
C2—S2—C1—S1	−179.63 (9)	C19—O1—C16—C17	176.42 (17)
C1—S2—C2—C3	177.34 (11)	C14—C15—C16—O1	178.67 (16)
S2—C2—C3—C8	−60.33 (18)	C14—C15—C16—C17	0.4 (3)
S2—C2—C3—C4	120.25 (14)	O1—C16—C17—C18	−178.96 (15)
C8—C3—C4—C5	−0.7 (2)	C15—C16—C17—C18	−0.6 (3)
C2—C3—C4—C5	178.73 (15)	C16—C17—C18—C13	0.3 (3)
C3—C4—C5—C6	1.1 (3)	C14—C13—C18—C17	0.1 (3)
C4—C5—C6—C7	−1.0 (3)	C12—C13—C18—C17	179.30 (16)
C5—C6—C7—C8	0.5 (3)		

Symmetry code: (i) −*x*+1, *y*, −*z*+1/2.

### Hydrogen-bond geometry (Å, º)

**Table:**

*D*—H···*A*	*D*—H	H···*A*	*D*···*A*	*D*—H···*A*
C10—H10*C*···N1^ii^	0.98	2.62	3.575 (2)	166

Symmetry code: (ii) −*x*+1, −*y*+2, −*z*+1.
